# Three-year assessment of cognitive and olfactory disturbances among COVID-19 convalescent patients grouped by olfactory hallucination status in Armenia: A qualitative and quantitative study

**DOI:** 10.1016/j.clinme.2025.100489

**Published:** 2025-07-16

**Authors:** Karine Melkumyan, Syuzanna Simonyan, Darshan Shingala, Hrag Torossian, Karen Mkrtumyan, Milena Tulbenjyan, Yekaterina Hovhannisyan, Konstantin Yenkoyan

**Affiliations:** a Department of Physiology, Yerevan State Medical University named after Mkhitar Heratsi, Yerevan 0025, Armenia; b COBRAIN Center, Yerevan State Medical University named after Mkhitar Heratsi, Yerevan 0025, Armenia; c Neuroscience Laboratory, COBRAIN Center, Yerevan State Medical University named after Mkhitar Heratsi, Yerevan 0025, Armenia; d Krisp Technologies Inc., Yerevan 0033, Armenia; e General Medicine Faculty, Yerevan State Medical University named after Mkhitar Heratsi, Yerevan 0025, Armenia; f Neurology Service at Neurosurgery Comprehensive Stroke Center, Heratsi N1 University Clinic, Yerevan State Medical University named after Mkhitar Heratsi, Yerevan 0025, Armenia

**Keywords:** SARS-CoV-2, Long-COVID, Olfactory distortions, Cognitive impairments, Depression

## Abstract

**Background:**

Smell disturbances, memory and mood changes are frequently reported as symptoms of long COVID that can be debilitating and long-lasting, having a detrimental impact on a patient’s quality of life and possibly contributing to depression and a decline in cognitive abilities.

**Study objective:**

This study aims to investigate long-term post-COVID cognitive and olfactory disturbances among the COVID-19 convalescent adult Armenian population aged between 18 and 65 years. The assessment extends to the differentiation of various olfactory distortions and association between various olfactory and cognitive variables, grouped by participants’ olfactory hallucination status.

**Study design:**

Explanatory sequential mixed-methods design was employed. Through three follow-up visits, the quantitative phase evaluated olfactory and cognitive abnormalities following COVID-19, comparing those with and without olfactory hallucinations. Through in-depth interviews, the qualitative phase investigated how participants perceived these symptoms and their impact on their quality of life.

**Study participants:**

The quantitative study participants were those who self-reported subjective disturbances in the olfactory perception 14 days following a COVID-19 diagnosis, as confirmed by a positive PCR test at the time of diagnosis. The qualitative study participants were those who self-reported persistent olfactory disturbances post-visit 3.

**Results:**

The study found that olfactory hallucinations lead to more pronounced depression compared with non-hallucinogenic types of olfactory disturbances. It was determined that a significant predictor of parosmia is persistent anosmia up to 4 months following COVID-19 infection.

**Conclusion:**

The long-term olfactory disturbances post-COVID-19 infection have a better prognosis among participants without olfactory hallucination compared to participants with olfactory hallucination.

## Highlights

• Hyposmia showed faster recovery, while parosmia took over 3 years.
• Persistent anosmia up to 4 months post-COVID predicts parosmia development.
• Phantosmia recovery was slow, with symptoms persisting for over 3 years.
• Olfactory hallucinations linked to more severe depression than non-hallucinatory disturbances.
• Olfactory memory decline correlates with cognitive impairments and heightened depression.

## Graphical abstract

**Figure:**
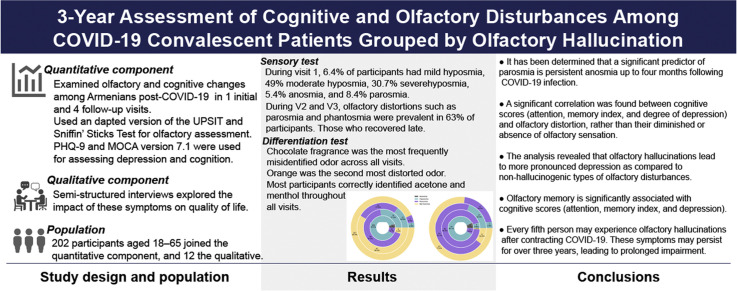

## Introduction

Olfactory and gustatory abnormalities are commonly observed in viral upper respiratory tract infections, including COVID-19 ^1^. During the acute phase of COVID-19, olfactory dysfunction affects 40–50% of patients, with up to 98% exhibiting abnormalities in objective smell and taste tests. Persistent dysfunction occurs in 10–20% of patients, affecting millions worldwide.^2^,^3^ These disturbances are categorised into quantitative or qualitative types.^4^ Quantitative disturbances include anosmia (complete loss of smell) and hyposmia (reduced smell perception), while qualitative disturbances include parosmia (distorted perception of actual odours) and phantosmia (perception of odours without any external stimulus). These qualitative disturbances can be particularly distressing and may persist for months after recovery.^5^ For instance, 20% of patients experience lasting olfactory dysfunction 1 year after the onset of COVID-19,^6^ while Ercoli *et al* (2021) found that 35% of patients had qualitative disturbances 3 months after infection.^7^ Furthermore, olfactory dysfunction varies across different populations and is influenced by cultural and sensory characteristics.^8^ Persistent olfactory disruptions are associated with depression and cognitive impairment. Research indicates that these issues may continue for over a year, significantly impacting affected individuals’ quality of life and mental health.^9^, ^10^, ^11^, ^12^ However, the causal relationships between these factors remain unclear. Investigating the olfactory disturbances requires the use of quantitative data, while understanding the impact that they have on the quality of life and mental health requires collecting qualitative data from the participants, because the same olfactory disturbance may differently affect the mental health of different participants. Hence, a mixed-methods design was the most appropriate for understanding the relationship between both. This study aims to explore post-COVID olfactory deviations in the Armenian population (quantitative component) and their associations with cognitive and emotional health (qualitative component).

## Materials and methods

### Study design

This study employed an explanatory sequential mixed-methods design, starting with quantitative data collection and analysis, followed by qualitative exploration. Quantitatively, it examined olfactory and cognitive disturbances among Armenians post-COVID-19, comparing olfactory improvement between participants who endured olfactory hallucinations (phantosmia, hereafter referred to as P1) and those who did not (P2). A longitudinal cohort design was implemented, starting with an initial assessment (visit 1, V1) and followed by three additional follow-ups (V2, V3 and V4) conducted from October 2020 to March 2023 by the COBRAIN Center.^13^ Participants reported changes in smell perception during follow-ups. Qualitatively, we followed the content analysis methodological orientation through conducting semi-structured interviews to explore the impact of these symptoms on quality of life (see Appendix A for the guide).

### Study setting and participants

Participants in the quantitative component were Armenians aged 18–65 with persistent post-COVID-19 olfactory disturbances. Exclusions included comorbidities affecting smell, negative anti-SARS-CoV-2 antibodies, or prior olfactory issues. For the qualitative component, participants were selected after V3 to capture their perspectives on symptom impact and coping strategies.

The quantitative component included participants of Armenian origin with persistent olfactory disturbances post-COVID-19 infection post-V1. The target population was native adult Armenians who self-reported subjective disturbances in the olfactory perception 14 days following a COVID-19 diagnosis, as confirmed by a positive PCR test at the time of diagnosis. Considering the effects of ageing on olfactory function, the age limit for inclusion in the study was determined to be from 18 to 65 years inclusive. Participants with negative anti-SARS-CoV-2 antibodies and those admitted to polyclinics due to complications were excluded from the study. Participants with a self-reported known history of comorbidities such as active allergies, acute rhinitis, neurodegenerative disorders etc, and the presence of olfactory disturbances prior to COVID-19 diagnosis due to other known causes such as recent rhinoplasty, trauma etc, were excluded from the study. Regarding the qualitative study component, participants with persistent olfactory disturbances post-V4 were separately interviewed face to face once without repetition by author Karine Melkumyan PhD, associate professor with 2 years of training in human psychology, to obtain a broad spectrum of perspectives on the impact of these chemosensory alterations on their improvement, quality of life and coping strategies utilised. All interviewed participants had also previously undergone the quantitative assessment and had known the interviewing researcher and the goal and interest of the study for 3 years.

### Study instruments

Quantitative instruments included an adapted version of the UPSIT and Sniffin’ Sticks Test^14^ (Appendix B) to assess odour identification. Each participant was assessed based on odour identification for a total of 16 odours across all visits, categorised as identified (1 point), unidentified (0) and wrongly identified (−1) for each odour. This test used a scale that yields a score between −16 and +16, resulting in a 32-point deviation system. Cognitive function was assessed using MoCA version 7.1^15^ (Appendix C), with severity thresholds. PHQ-9^16^ was used for depression screening, scoring severity from mild to severe. The PHQ-9 score ≥10 had a sensitivity of 88% and a specificity of 88% for major depression^16^ (Appendix D).

### Participants’ sampling and data collection

Quantitative data included 202 participants and were collected from October 2020 to March 2023, including participants with persistent symptoms across V2, V3 and V4, achieving a 100% response rate. For qualitative analysis, a follow-up phone call was made to the participants after 1 year of V4. Those who still had disturbances were invited for an interview. A total of 12 in-depth interviews were conducted from May 2024 to July 2024 using purposive sampling to explore themes emerging from quantitative findings. Interviews averaged 30 min, were conducted at the COBRAIN Center, audio recorded, translated and transcribed by the authors who spoke the native language of the participants, and therefore were not sent for feedback from the participants. Data collection ceased upon reaching saturation and the transcriptions were accordingly analysed.

### Data analyses and study rigor

Quantitative data were analysed using IBM SPSS software 26^17^ and analysed using Python 3.9.7^18^ and Stata Corp. 2013,^19^ including all descriptive statistics (frequency, mean and standard deviation), chi-square for group comparisons of categorical variables, and independent and one-sample *t*-tests for group comparisons of continuous variables. Fisher’s exact test was used instead of the chi-square test if an expected cell count was less than 5. The data are presented using counts and percentages. The normally distributed demographic variables are presented with means and standard deviations (SDs). Analyses were conducted on the entire sample of 202 participants across all four visits and on two subgroups – P1 ‘with phantosmia’ and P2 ‘without phantosmia’ groups. Missing data were imputed using kNN (k-nearest neighbour). Thematic analysis of qualitative data identified patterns and categories through iterative coding done collectively by the authors using MS Word 2019. Findings from quantitative data were contextualised using qualitative themes, ensuring comprehensive results.

### Correlation analysis

A Pearson point-biserial correlation analysis was used to examine associations between dichotomous and continuous olfactory parameters. In addition, a heatmap correlation matrix was generated to visualise the relationships among olfactory, cognitive and mood-related variables, including PHQ and MoCA scores. The matrix was constructed using Python 3.9.7 package and is presented in Appendix F (Fig. F.1).

## Results

### Quantitative

#### Descriptive analysis

##### Assessment of olfactory perception

A total of 202 participants met the inclusion/exclusion criteria for V1, with a mean age of 37.04 ± 11.82 years (range: 18–65). Of these, 74.75% (151 participants) were female and 25.25% (51 participants) were male. Sensory tests showed that 6.4% had mild hyposmia, 49.0% moderate hyposmia, 30.7% severe hyposmia, 5.4% anosmia and 8.4% parosmia.

During V2 and V3, olfactory distortions such as parosmia and phantosmia were prevalent in 63.0% of participants, particularly those with slower recovery post-COVID-19. These distortions, emerging 2–4 months post-diagnosis (primarily in the third month), were more common among those with prolonged convalescence.

Considering this finding, participants were further divided into two groups based on the presence or absence of olfactory hallucination (phantosmia), which are denoted as group P1 (with phantosmia) and group P2 (without phantosmia) from now onwards.

The V1 sensory tests for P1 participants revealed that 85.1% of the participants had hyposmia, 6.9% had anosmia and 8% had parosmia. The V1 sensory tests for P2 participants revealed that 87.0% of the participants had hyposmia, 4.3% had anosmia and 8.7% had parosmia.

**Fig 1:**
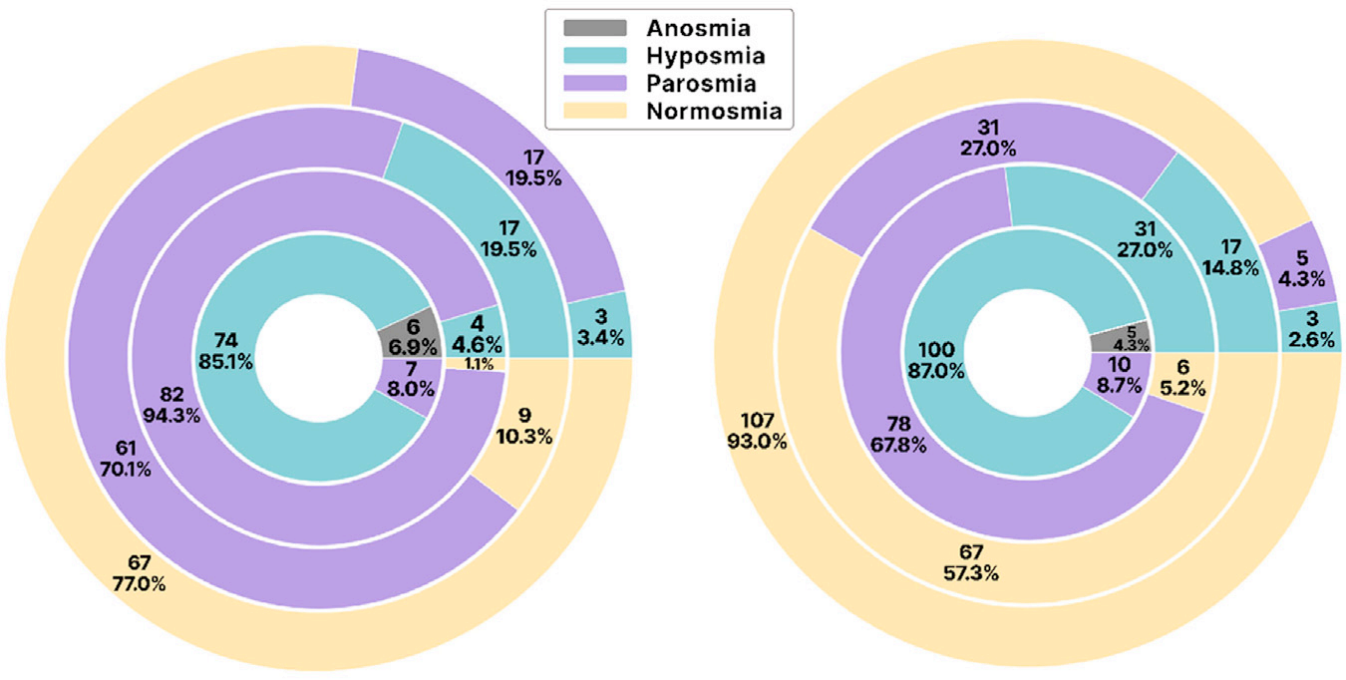
Quantitative distribution of olfactory disturbances among P1 (Left) and P2 (right) during all four visits. Visits 1–4 are represented in order from smaller to larger circles.

In V2, 202 participants were included. Among them, 87 (43.1%) experienced phantosmia, while 115 (56.9%) did not. In V3, parosmia was reported by 70.1% of P1 participants, while 58.3% of P2 participants had fully recovered. V4 sensory tests showed significant improvement in the P2 group compared to the P1 group (see Fig. 1).

**Fig 2:**
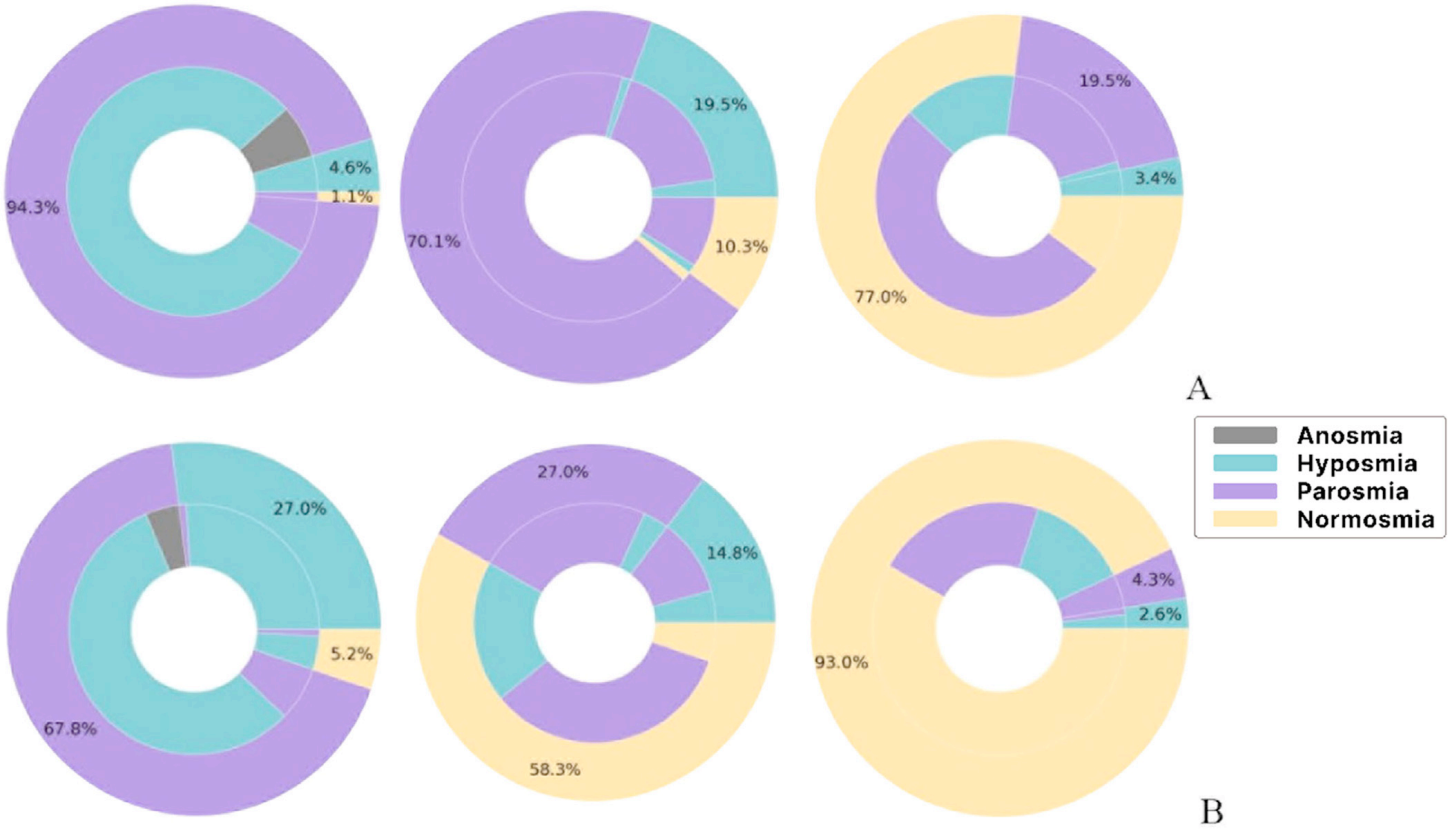
Status of olfactory disturbance at each subsequent visit (outer region) versus the previous visit among P1 (A) and P2 (B).

The analysis also revealed that both P1 and P2 participants categorised to have anosmia in V1 were assessed to have olfactory distortions in V2. It also revealed that the majority of participants with anosmia during V1 developed parosmia during V2. It was also noted that only the participants who encountered olfactory distortion in the previous visit came for the follow-up visits. There was no drop-out between visits. The juxtaposition of olfactory distortions perception of participants between visits among P1 and P2 participants is represented in Fig. 2A and B, respectively.

Analysis revealed significant differences in improvement rates between P1 and P2 participants with hyposmia during V2 (*p* = 0.030). P2 participants with hyposmia in V1 recovered faster across subsequent visits compared to P1 participants (*p* < 0.000). Nearly all P1 participants with hyposmia in V1 developed parosmia by V2, with improvement observed from V3 onward.

Longitudinally, 28 of 202 participants (13.9%) showed no olfactory improvement after 3 years, with 20 from P1 and eight from P2.

In both the P1 and P2 participants, the olfactory perception was assessed to be consistently recovering at approximately 20.0% for 3 years post-severity peak in V2.

The olfactory test showed that chocolate fragrance was the most frequently misidentified odour across all visits. In V1 and V2, 180 and 160 participants, respectively, failed to identify the aroma, while five and 53 participants recognised it incorrectly. By V3 and V4, the number of participants misidentifying chocolate decreased to 71 and eight, respectively.

In contrast, most participants correctly identified acetone and menthol throughout all visits. Orange fragrance was the second most distorted odour, with 109, 46, 15 and six participants misinterpreting it in V1, V2, V3 and V4, respectively (Appendix E).

The analysis suggests that the P1 participants significantly misidentified all 16 odours individually as well as combined in comparison to P2 participants across all visits (*p*-value < 0.004).

##### PHQ

The PHQ assessment revealed that depressive symptoms peaked during V2, with P1 participants showing significantly higher PHQ scores compared to P2 participants (*p*-value < 0.013). Higher PHQ scores reflect greater severity of depressive symptoms. Among P2 participants, mean PHQ scores were 7.7, 9.8, 7.5 and 4.3 for V1, V2, V3 and V4, respectively, while P1 participants had mean scores of 11.0, 14.3, 10.8 and 7.0 during the same visits.

**Fig 3:**
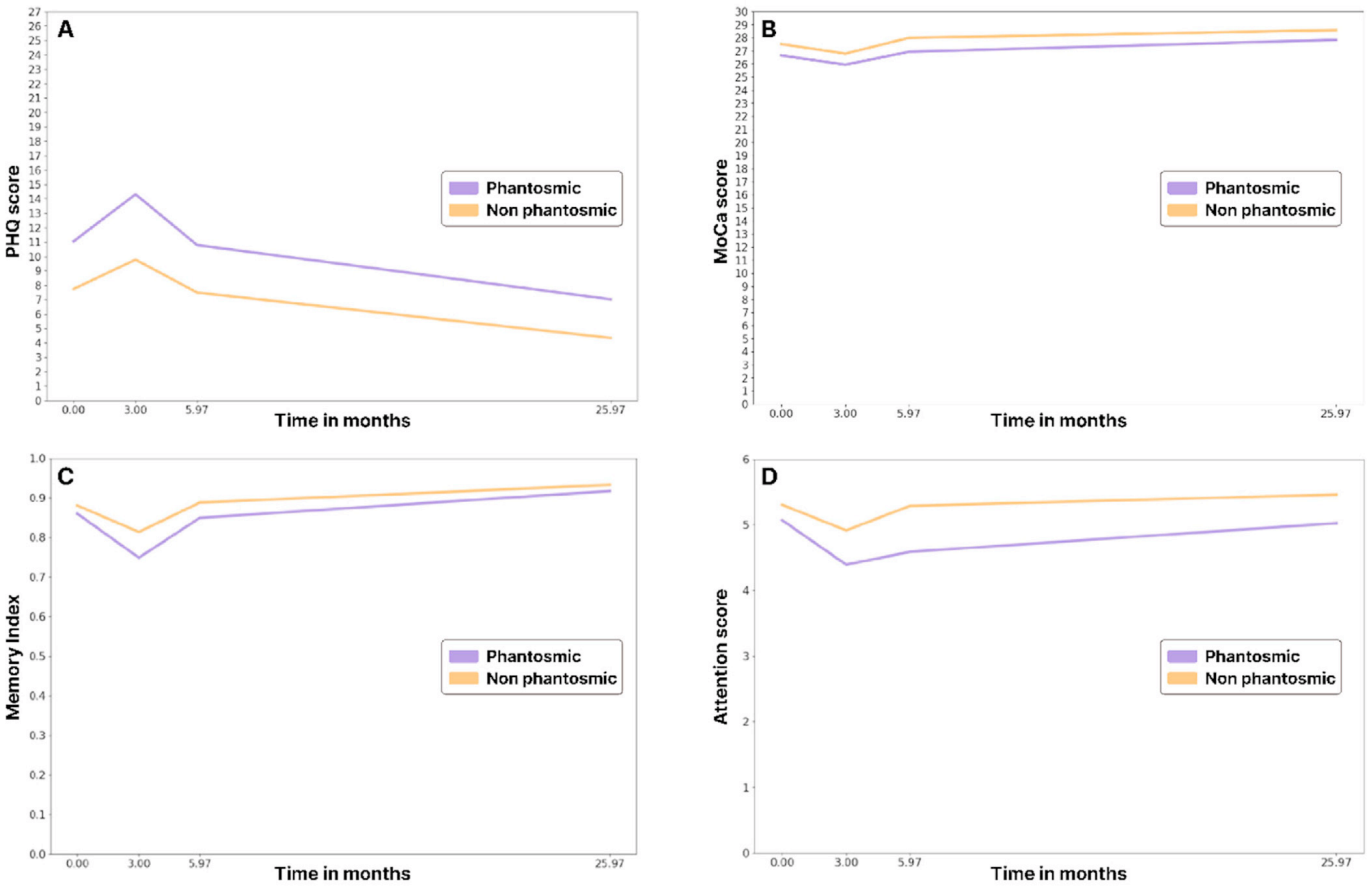
Changes in the mean values of PHQ-9 mean scores (A), MoCA test total (B), and components: memory index (C) and attention (D) in the two groups of the study in the dynamics of four visits.

The relative risk of developing depressive symptoms was 30.0–40.0% higher in P1 participants compared to P2 participants. See Fig. 3A for graphical representation.

##### MoCA

Cognitive functioning assessed through the MoCA test revealed that scores were lowest during the second visit, particularly in the P1 group. Subdomains such as attention and short-term memory were more significantly impaired compared to other areas like visuospatial/executive function, language and orientation. These effects were markedly more prevalent among P1 group participants (Fig. 3B, C, D).

##### Correlations

Significant correlations were observed between cognitive and depressive signs and the presence of olfactory distortion. Specifically, attention, memory and depression scores were also associated with participants’ ability to identify odours without hearing the options. These associations are visualised in the correlation heatmap provided in Appendix F (Fig. F1).

### Qualitative

Twelve participants (11 female and one male, aged 25–42) were interviewed from March to May 2024. Using a semi-structured interview guide, seven major themes and 24 subthemes were identified (see Appendix G for detailed descriptions and supporting quotes). Below is a summary of each theme:

Theme 1: Olfactory disturbances. Participants experienced various changes in their sense of smell, including anosmia, hyposmia, parosmia and phantosmia. This matched the quantitative findings, where 17 participants (19.5%) reported parosmia during V4 and confirmed these experiences during follow-up interviews 1 year later.

Theme 2: Gustatory disturbances. Taste changes often mirrored olfactory distortions. Familiar foods became unpleasant or even ‘disgusting’, leading to decreased food intake, weight loss and lower quality of life, consistent with increased PHQ scores in the quantitative data.

Theme 3: Timeline of chemosensory alteration. Participants reported diverse trajectories of symptom progression – sudden, gradual, non-linear or persistent. Most participants experienced a transition from initial anosmia to hyposmia, while those who developed parosmia tended to have prolonged symptoms that persisted through V3 and V4.

Theme 4: Severity of chemosensory alteration. The intensity of olfactory and gustatory dysfunction ranged from mild to severe. Severe cases significantly disrupted daily activities and mental health, as reflected in PHQ-9 and MoCA scores.

Theme 5: Impact on quality of life. Chemosensory disturbances affected multiple domains – physical, emotional, social and psychological. Participants reported depression, anxiety, strained relationships and, in extreme cases, suicidal ideation. These findings paralleled elevated PHQ-9 scores and cognitive complaints noted in MoCA assessments. A participant went as far as saying that the worst consequence of COVID was that they began to smell their child differently.

Theme 6: Coping mechanisms. Participants adopted various strategies to manage symptoms, including medical treatment, self-medication, olfactory training, and lifestyle changes aimed at minimising the impact of sensory loss.

Theme 7: Perceptions pre- and post-COVID. Participants described how their perceptions of health and sensory function evolved over time. Many expressed surprises at the persistence of symptoms, which lasted over a year and significantly altered their daily experiences and self-perception. expressed that the effects were often unanticipated and persisted for over a year after their initial onset.

In summary, the thematic analysis in Table G.1 offers rich context for interpreting the quantitative findings. Notably, parosmia emerged as a significant predictor of long-term chemosensory impairment, while phantosmia was often described as the most distressing and persistent symptom. Together, the results emphasise the need for targeted interventions addressing prolonged olfactory and cognitive disturbances in COVID-19 convalescent patients.

## Discussion

A unique strength of this study is the 3-year longitudinal follow-up of 202 patients using both quantitative and qualitative approaches to assess olfactory outcomes and their psychological correlates. Our findings reveal that patients with olfactory distortions such as parosmia and phantosmia had significantly prolonged recovery compared to those with hyposmia alone. This aligns with previous literature, which has reported slower resolution of qualitative olfactory dysfunctions.^20^ Notably, participants with phantosmia had the slowest recovery trajectories, and this group also reported the highest levels of depression. This supports growing evidence that olfactory hallucinations may exert greater psychological burden than simple sensory loss.^21^ Additionally, 17 out of 84 participants continued to experience olfactory difficulties even 3 years after COVID-19, suggesting long-term quality of life impairments. The association between prolonged olfactory dysfunction and depressive symptoms highlights the importance of integrated sensory and mental health support in post-COVID care. Compared to prior work, our inclusion of both subjective reports and clinical follow-up over an extended period adds to understanding the trajectory and impact of COVID-19-related olfactory dysfunctions.^13^

The research indicates that COVID-19-related olfactory disturbances may persist for years, with a clear trend showing that symptoms which do not improve within the first 10 days tend to become chronic. This finding in the literature closely aligns with our data, suggesting that the median recovery time for most patients (93%) is 14 days.^22^

Tiana Saak and colleagues also observed that persistent olfactory dysfunction linked to COVID-19 carries a significant risk of developing neuropsychiatric disorders, including depression and anxiety, even nearly 2.5 years after the initial COVID-19 diagnosis.^23^ These findings emphasise the need for continued monitoring, early intervention and potential psychological support for affected individuals.

The purpose of interviewing participants with phantosmia was to determine the quality changes in their lives brought on by prolonged exposure to COVID-19. Participants lacked drive, enthusiasm and confidence to work, as shown in Table G, and they even lost weight when they gave up their favourite foods because of the unpleasant odour. One of the participants even considered suicide. Additionally, we found that the emergence of undesirable fragrances is a key factor in depression, compounded by the deprivation of beloved foods and scents. This ultimately contributes to apathy towards life, as reflected in declines in memory and concentration.

Similar results are presents in the literature, indicating that individuals who suffer from lasting qualitative olfactory dysfunction have limitations in daily life and impaired eating patterns.^21^ However, a high proportion of female participants included in this study is considered a limitation and requires a study with balanced gender proportion to understand gender-based differences.

Memory and attention were the two cognitive functions that were most negatively impacted. Furthermore, the high correlation of memory deterioration with phantosmic group allows us to assume that central lesions play a particularly crucial role in the development of phantosmia. The participant once more seemingly ‘learns’ to distinguish odours that are now novel to them after their perception of the fragrance is erased. This is strengthened by the significant association found between memory decline in participants with persistent phantosmia.

This idea is supported by Alberto Arrigoni and colleagues, who observed potential substantial changes in brain microstructure, morphology and neuronal connectivity in COVID-19 patients experiencing olfactory disturbances and cognitive dysfunction.^24^

Participants were not the same as they were prior to the illness. This was evidenced by the subjective feelings expressed in their interviews, such as how they miss the smell perception from their routine life.

The study used a convenience sampling approach by inviting participants with persistent olfactory disturbances for follow-up, which might introduce selection bias. Those with milder or resolved symptoms may have been underrepresented, potentially skewing the findings toward more severe cases. Although the study instruments (eg UPSIT, MoCA, PHQ-9) are validated, their adaptation to the Armenian context may have introduced cultural nuances that were not fully accounted for. The prolonged study period (2020–2024) encompassed varying phases of the pandemic, potentially confounding results due to changing healthcare practices, public health interventions, and variants of the SARS-CoV-2 virus.

Over the past 3 years of study, we observed that long-term olfactory disturbances following COVID-19 have a better prognosis in cases of hyposmia. In contrast, olfactory distortions appear to be a major predictor of persistent impairment, suggesting that full recovery may not always be possible.

Similar findings have been reported in the literature, indicating that majority of patients who initially experienced smell and taste disturbances did not achieve full recovery even after 2 years’ follow-up.^25^To further investigate this, we will continue monitoring the 17 patients who still report symptoms.

These clinical observations are further contextualised by our previous studies, which demonstrated changes in the kinetics of SARS-CoV-2-specific antibodies^26^, ^27^, their age-related dynamics^28^ and distinct genetic correlates within the Armenian population^29^ that may influence long-term immune and neurological outcomes. Together, these findings underscore the complex and multifactorial nature of post-COVID sequelae – including persistent olfactory disturbances – shaped by immune, genetic and neuropsychiatric interactions.

### Future applications and international implications

Our findings have important translational implications. First, the identification of olfactory hallucinations (especially phantosmia) as both a psychological stressor and a potential early marker of cognitive decline provides a cost-effective avenue for screening individuals at risk of long COVID-related neurocognitive impairment. Brief and inexpensive smell tests (eg Sniffin’ Sticks or UPSIT) administered in primary care settings, combined with rapid cognitive screening tools like MoCA, may enable early detection and targeted intervention in resource-limited environments. This approach is particularly feasible given the non-invasive, scalable and low-cost nature of these assessments.

The neurocognitive tests used in our study were chosen based on their established sensitivity to the domains most affected by long COVID – working memory, attention and executive function – and their prior use in neurological and psychiatric evaluations. If adapted for international implementation, we propose maintaining the core cognitive domains while integrating culturally and linguistically validated equivalents. Frameworks such as the NIH Toolbox or the Montreal Cognitive Assessment (MoCA) offer validated translations and are adaptable to multicentre protocols. Harmonisation across centres would ensure consistency while allowing population-specific adjustments to preserve cross-cultural relevance.

These results underscore the need for global, longitudinal and interdisciplinary collaborations to develop streamlined post-COVID screening protocols. Integrating olfactory and cognitive assessments into long COVID care pathways could significantly improve early identification and intervention for at-risk individuals.

## Conclusion

Our study highlights the complex and long-lasting impact of COVID-19 on olfactory and cognitive functions. We identified persistent anosmia up to 4 months post-infection as a significant predictor of parosmia. Furthermore, olfactory distortions, rather than a mere reduction or absence of smell, were more strongly correlated with cognitive impairments, including deficits in attention, memory and depression severity. Notably, olfactory hallucinations were associated with more pronounced depressive symptoms compared to other types of olfactory disturbances. Our findings also indicate that olfactory memory plays a crucial role in cognitive function, with its impairment contributing to diminished odour perception. Longitudinal data suggest that approximately one in five individuals may experience olfactory hallucinations after COVID-19, with symptoms persisting for over 3 years, raising concerns about potential long-term olfactory dysfunction. These findings underscore the need for further research into the underlying mechanisms and the development of targeted interventions to mitigate these effects.

## CRediT authorship contribution statement

**Karine Melkumyan:** Writing – review & editing, Writing – original draft, Visualization, Supervision, Resources, Project administration, Methodology, Investigation, Conceptualization. **Syuzanna Simonyan:** Writing – review & editing, Writing – original draft, Visualization, Validation, Resources, Methodology, Investigation. **Darshan Shingala:** Writing – review & editing, Writing – original draft, Validation, Software, Methodology, Formal analysis, Data curation. **Hrag Torossian:** Writing – review & editing, Writing – original draft, Visualization, Validation, Software, Methodology, Investigation, Formal analysis, Data curation. **Karen Mkrtumyan:** Software, Formal analysis, Data curation. **Milena Tulbenjyan:** Writing – review & editing, Writing – original draft, Visualization, Validation, Resources, Investigation. **Yekaterina Hovhannisyan:** Visualization, Validation, Resources. **Konstantin Yenkoyan:** Conceptualization, Methodology, Validation, Resources, Writing – original draft, Writing – review & editing, Visualization, Supervision, Funding acquisition.

## Declaration of competing interest

The authors declare that they have no known competing financial interests or personal relationships that could have appeared to influence the work reported in this paper.

## Appendix Supplementary materials

Image, application 1

Image, application 2

Image, application 3

Image, application 4

Image, application 5

Image, application 6

Appendix A. Semi Structured Interview Guide (English & Armenian + Consent Form). Image, application 7

## References

[1] Neta FI., Fernandes ACL., Vale AJM. (2021). Pathophysiology and possible treatments for olfactory-gustatory disorders in patients affected by COVID-19. Curr Res Pharmacol Drug Discov.

[2] Frasnelli J., Tognetti A, Winter AL., Frasnelli J. (2024). High prevalence of long-term olfactory disorders in healthcare workers after COVID-19: a case-control study. PLoS One.

[3] Lenz C., Slack M.P.E., Shea K.M., Reinert R.R., Taysi B.N., Swerdlow D.L. (2024). Long-Term effects of COVID-19: a review of current perspectives and mechanistic insights. Crit Rev Microbiol.

[4] Espetvedt A., Wiig S., Myrnes-Hansen K.V., Brønnick K.K. (2023). The assessment of qualitative olfactory dysfunction in COVID-19 patients: a systematic review of tools and their content validity. Front Psychol.

[5] Fageeh Y.A., Altuwaireqi A.S., Alghuraibi A.B., Alotaibi M.S., Alsulimany L.E., Altooarki E.A. (2024). Persistent smell disorders after COVID-19 infection and their impact on quality of life. Cureus.

[6] Boscolo-Rizzo P., Menegaldo A., Fabbris C. (2021). Six-month psychophysical evaluation of olfactory dysfunction in patients with COVID-19. Chem Senses.

[7] Ercoli T., Masala C., Pinna I. (2021). Qualitative smell/taste disorders as sequelae of acute COVID-19. Neurol Sci.

[8] Okrzeja J., Sołomacha S., Alimowski M. (2024). Assessment of smell disturbances 6 months after COVID-19 in Polish population. Sci Rep.

[9] Kohli P., Soler Z.M., Nguyen S.A., Muus J.S., Schlosser R.J. (2016). The association between olfaction and depression: a systematic review. Chem Senses.

[10] Speth M.M., Singer-Cornelius T., Oberle M., Gengler I., Brockmeier S.J., Sedaghat A.R. (2020). Mood, anxiety and olfactory dysfunction in COVID-19: evidence of Central nervous system involvement?. Laryngoscope.

[11] Sauer E.L., Venesky MD., McMahon TA. (2024). Are novel or locally adapted pathogens more devastating and why? Resolving opposing hypotheses. Ecol Lett.

[12] Grimshaw B., Chaudhuri E. (2021). Mental-health-related admissions to the acute medical unit during COVID-19. Clin Med.

[13] Melkumyan K., Shingala D., Simonyan S. (2022). Assessment of smell and taste disturbances among COVID-19 convalescent patients: a cross-sectional study in Armenia. J Clin Med.

[14] Doty R.L., Shaman P., Kimmelman C.P., Dann M.S. (1984). University of pennsylvania smell identification test: a rapid quantitative olfactory function test for the clinic. Laryngoscope.

[15] Nasreddine Z.S., Phillips NA., Bédirian V. (2005). The Montreal Cognitive Assessment, MoCA: a brief screening tool for mild cognitive impairment. J Am Geriatr Soc.

[16] Kroenke K., Spitzer R.L., Williams J.B.W. (2001). The PHQ-9: validity of a brief depression severity measure. J Gen Intern Med.

[17] IBM Corp. (2019). IBM SPSS Statistics for Windows, Version 26.0.

[18] Van Rossum G., Drake F.L. (2009).

[19] StataCorp (2013). Stata Statistical Software: Release 13.

[20] Boscolo-Rizzo P., Hopkins C., Menini A (2022). Parosmia assessment with structured questions and its functional impact in patients with long-term COVID-19-related olfactory dysfunction. Int Forum Allergy Rhinol.

[21] Winter A.L., Henecke S., Lundström JN., Thunell E.. (2023). Impairment of quality of life due to COVID-19-induced long-term olfactory dysfunction. Front Psychol.

[22] Zaid E.A., Eltelety A.M., Azooz K.O. (2024). Assessment of olfactory recovery after COVID-19: cross-sectional study. Eur Arch Otorhinolaryngol.

[23] Saak T.M., Tervo J.P., Vilarello B.J. (2024). Depression, anxiety, and neuropsychiatric symptom burden in a longitudinal cohort with persistent psychophysical post-COVID olfactory dysfunction. Brain Sci.

[24] Arrigoni A., Previtali M., Bosticardo S. (2024). Brain microstructure and connectivity in COVID-19 patients with olfactory or cognitive impairment. Neuroimage Clin.

[25] Boldes T., Ritter A., Soudry E. (2024). The long-term effect of COVID-19 infection on olfaction and taste; a prospective analysis. Eur Arch Oto-Rhino-Laryngol: Off J Eur Fed Oto-Rhino-Laryngol Soc (EUFOS): Affil Ger Soc Oto-Rhino-Laryngol - Head Neck Surg..

[26] Movsisyan M., Chopikyan A., Kasparova I. (2022). Kinetics of anti-nucleocapsid IgG response in COVID-19 immunocompetent convalescent patients. Sci Rep.

[27] Movsisyan M., Truzyan N., Kasparova I. (2024). Tracking the evolution of anti-SARS-CoV-2 antibodies and long-term humoral immunity within 2 years after COVID-19 infection. Sci Rep.

[28] Movsisyan M., Harutyunyan H., Movsisyan K. (2025). Age-related peculiarities of antibody-mediated humoral immune response following SARS-CoV-2 infection. Exp Gerontol.

[29] Hovhannisyan Y, Yeritsyan H, Hakobjanyan G (2025). Genetic Correlates of Presenile Dementia and Cognitive Decline in the Armenian Population Following COVID-19: A Case-Control Study. Int J Mol Sci.

