# Supplementary material for: Three-year assessment of cognitive and olfactory disturbances among COVID-19 convalescent patients grouped by olfactory hallucination status in Armenia: A qualitative and quantitative study
Source: Clin Med (Lond). 2025 Jul 16;25(5):100489. doi: 10.1016/j.clinme.2025.100489 (PMC12395519; doi:10.1016/j.clinme.2025.100489)
Supplement: Supplementary file 3 [file mmc3.docx]

**Appendix A – Semi Structured Interview Guide (English & Armenian + Consent Form)**

1. Could you please describe how COVID has influenced your life, and what has altered during and after the illness?

1.1 Can you recall what you were doing during the pandemic-induced isolation period, how your household, workplace, and rhythm of life were disrupted, and how you felt and behaved during that time? Could you please describe how COVID has influenced your life, and what has altered during and after the illness? Can you recall what you were doing during the pandemic-induced isolation period, how your household, workplace, and rhythm of life were disrupted, and how you felt and behaved during that time?

1.2 How has COVID impacted your thought processes function? Have you experienced difficulties thinking, remembering names, performing arithmetic, paying attention, concentrating, or perceiving new information while and after being sick with COVID? How has COVID impacted your initiative?

1.3 Describe the COVID disturbances that have occurred to date, how you are adapting to them, and what steps you have done and are taking to address the concerns.

1.4 Can you explain how olfactory distortions vary depending on the time of day, day of the week, your mood, and other factors?

2. Please try to describe in as much detail as possible all the changes in the sense of smell since the first day of COVID.

2.1 How did it all start, were the changes abrupt or did they gradually become more pronounced?

2.2 During these 2 years, was there a period when you noticed improvement, followed by deterioration again, or did things gradually improve?

2.3 Before getting sick with COVID, have you ever had smell problems due to various reasons: flu, cold, season, pregnancy...

3. Please talk about the changed smells. Which smells have changed and how have they changed?

3.1 Are all smells changed now, or are there any smells you smell exactly like before covid? Do you have to smell, do you have to have something close to your nose to get a distorted smell, or can you smell without smelling anything, when is this observed, what type of smell do you smell? for no reason.

3.2 Can you count all the smells that have been tainted at some point since COVID and identify those that are still tainted? Is the change similar, how would you describe it?

4. In addition to the smell problems, what other health problems did you have during COVID? Please describe the treatment process. If so, are there any health problems that arose during COVID and are still bothering you?
