# Supplementary material for: Three-year assessment of cognitive and olfactory disturbances among COVID-19 convalescent patients grouped by olfactory hallucination status in Armenia: A qualitative and quantitative study
Source: Clin Med (Lond). 2025 Jul 16;25(5):100489. doi: 10.1016/j.clinme.2025.100489 (PMC12395519; doi:10.1016/j.clinme.2025.100489)
Supplement: Supplementary file 4 [file mmc4.docx]

**Appendix B – The University of Pennsylvania Smell Identification Test (UPSIT) and Snip-And-Sniff Test**

**
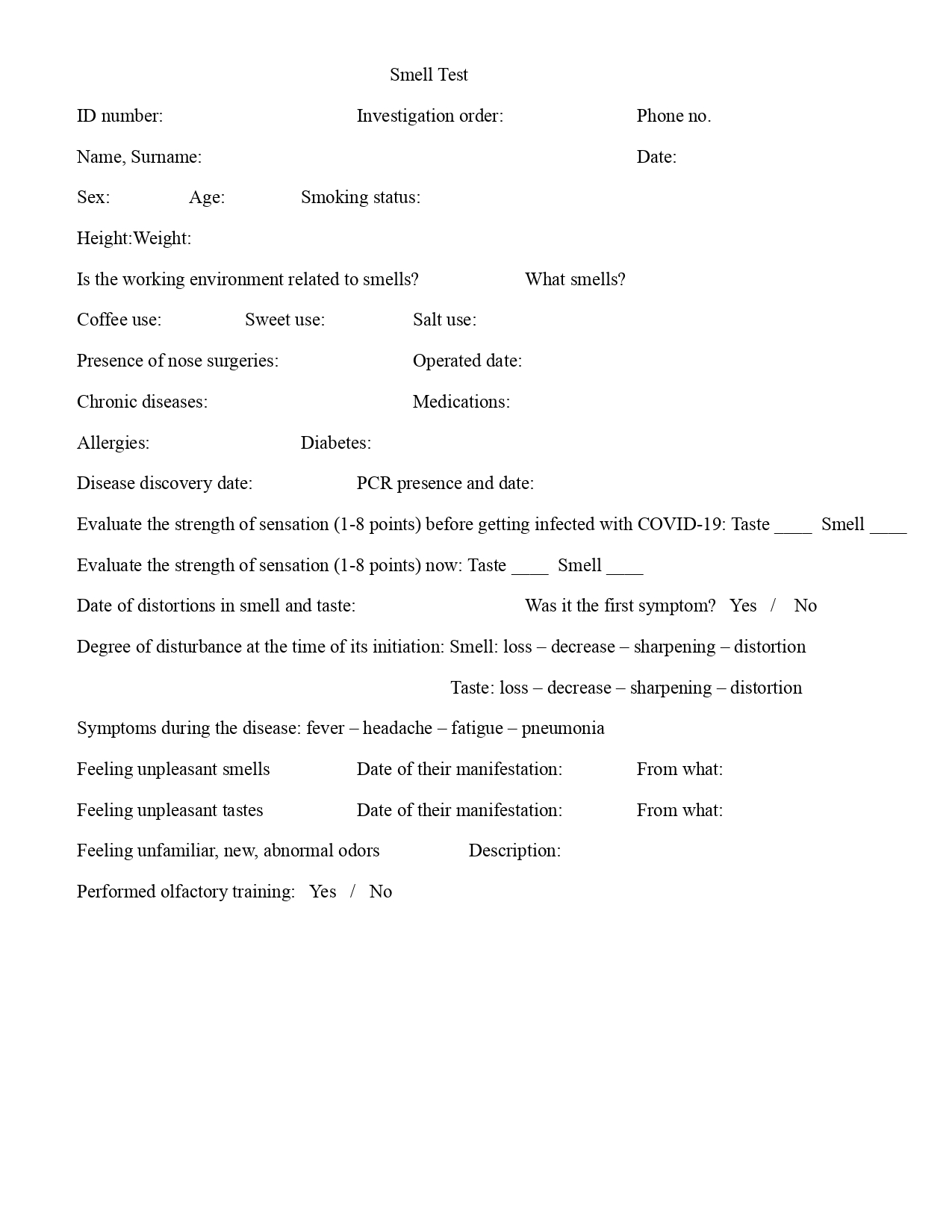
**

**
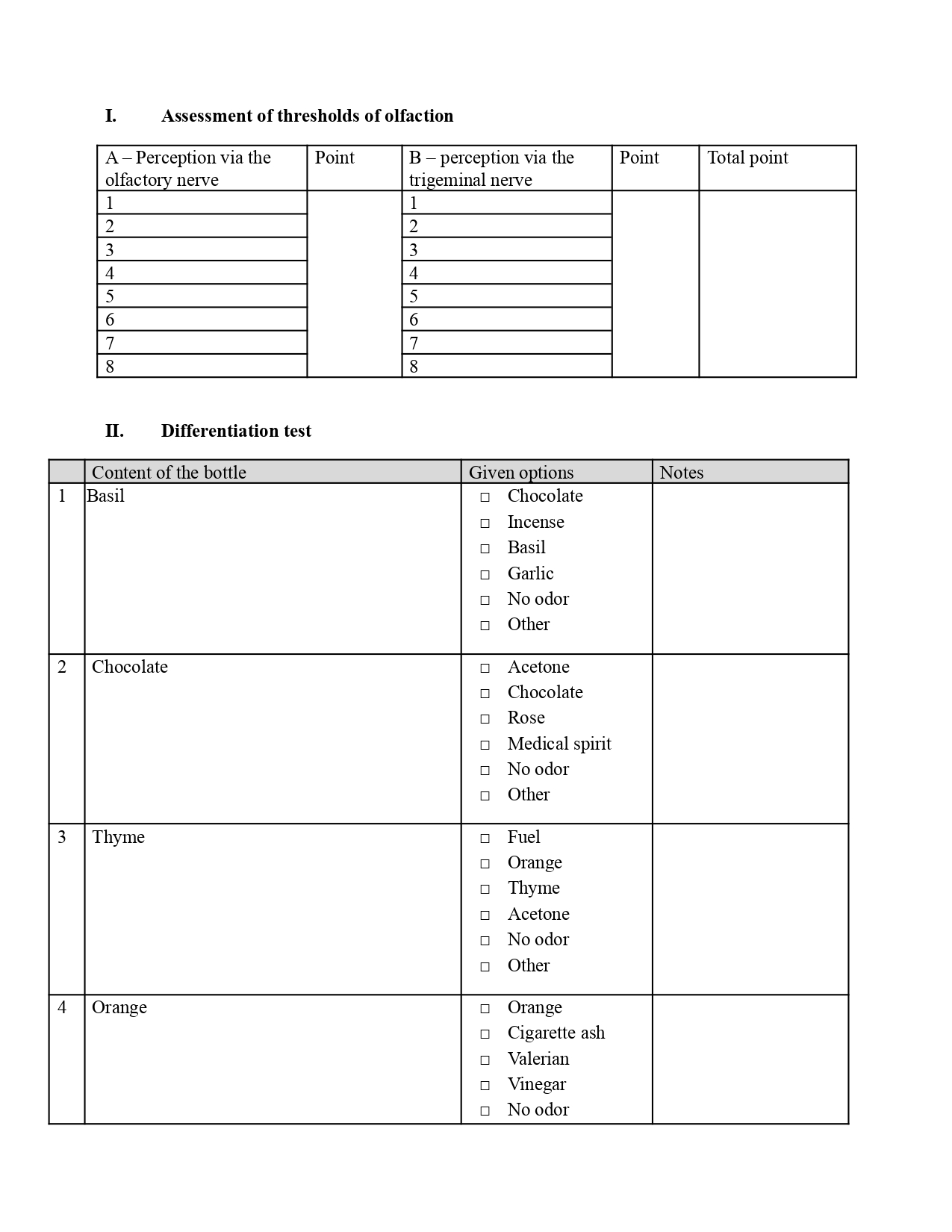
**

**
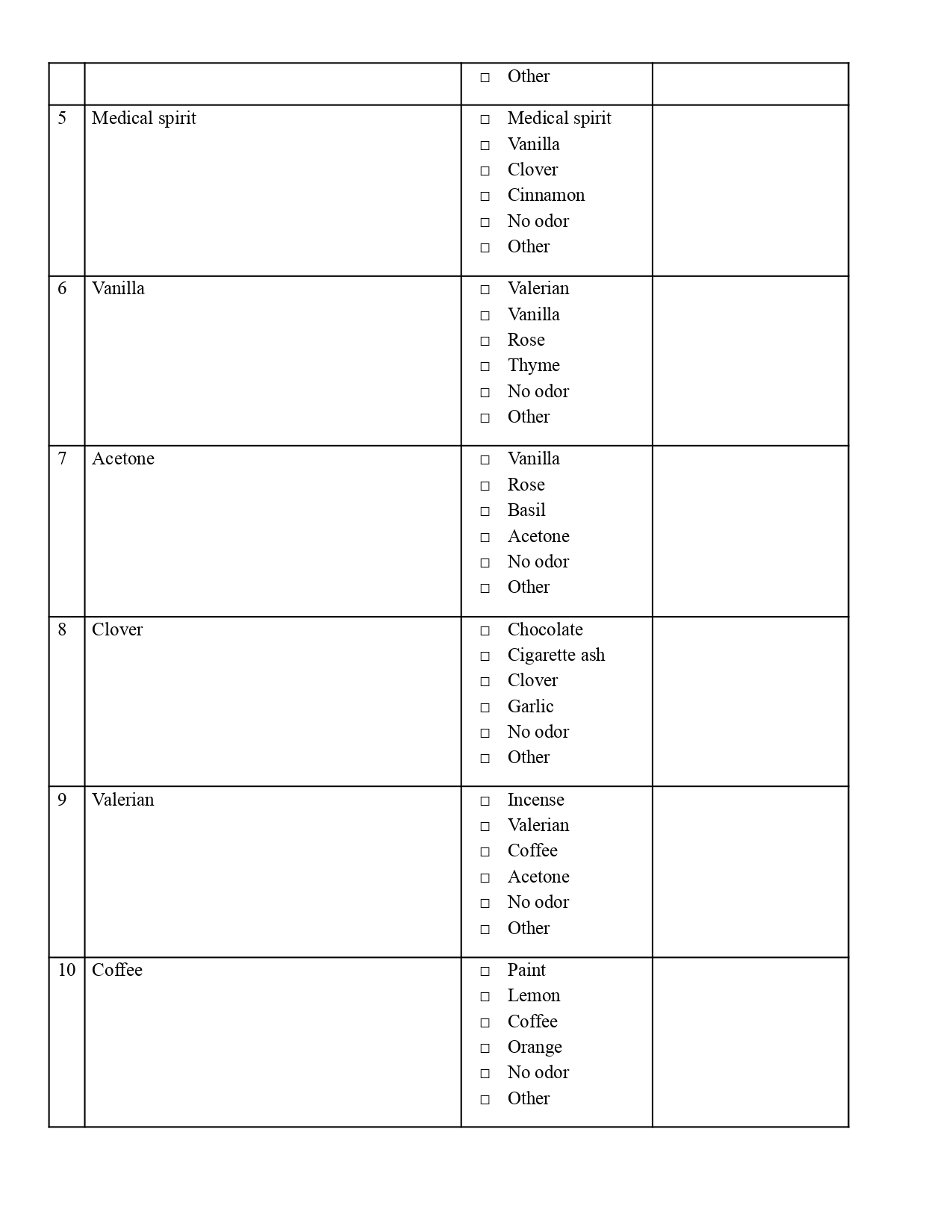
**

**
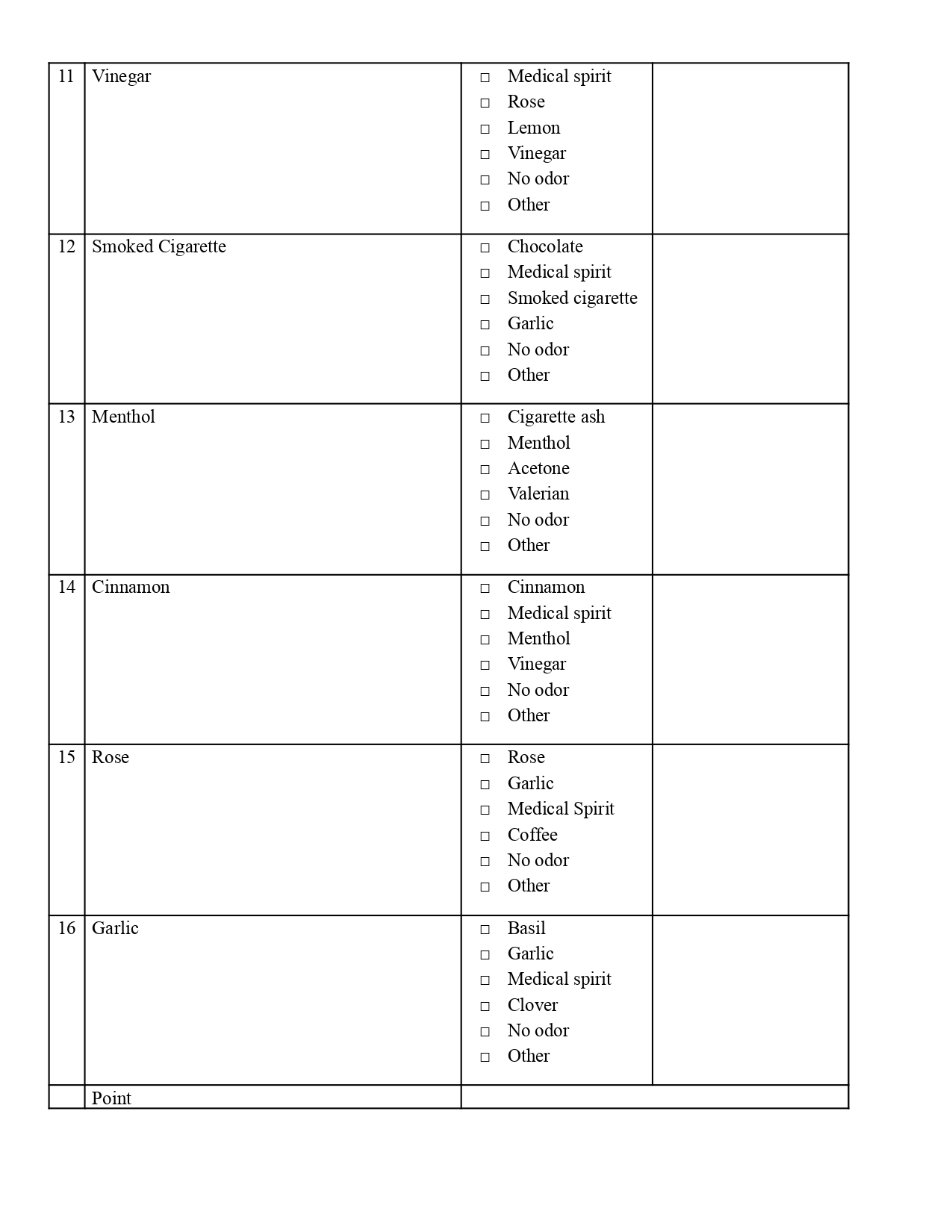

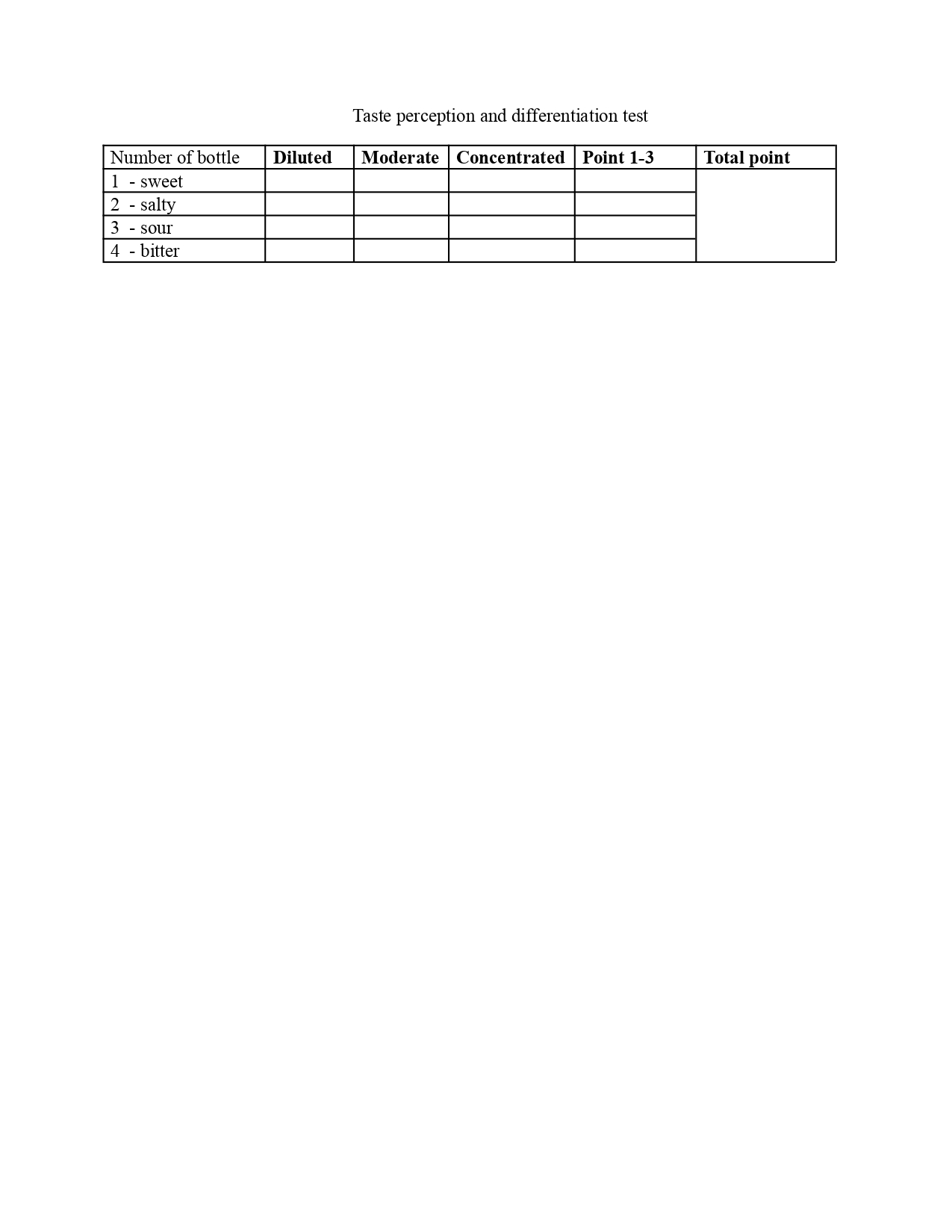
**
