# Supplementary figures and images for: Three-year assessment of cognitive and olfactory disturbances among COVID-19 convalescent patients grouped by olfactory hallucination status in Armenia: A qualitative and quantitative study
Source: Clin Med (Lond). 2025 Jul 16;25(5):100489. doi: 10.1016/j.clinme.2025.100489 (PMC12395519; doi:10.1016/j.clinme.2025.100489)

**Appendix C – Montreal Cognitive Assessment 7.1 (MOCA) - Armenian version**


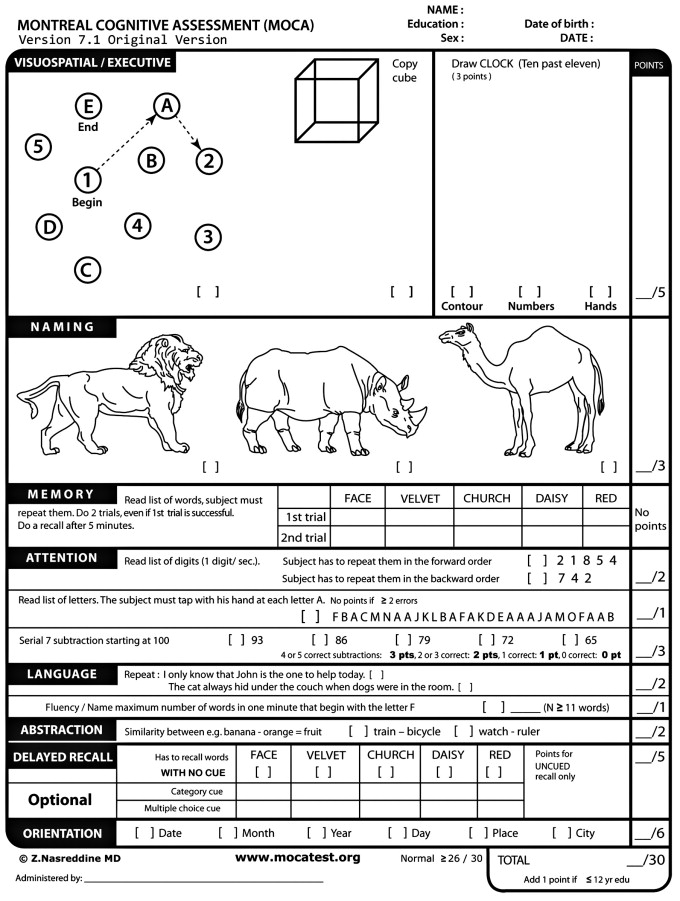

Supplement: Supplementary file 5 [file mmc5.docx]

**Appendix D – PHQ-9**


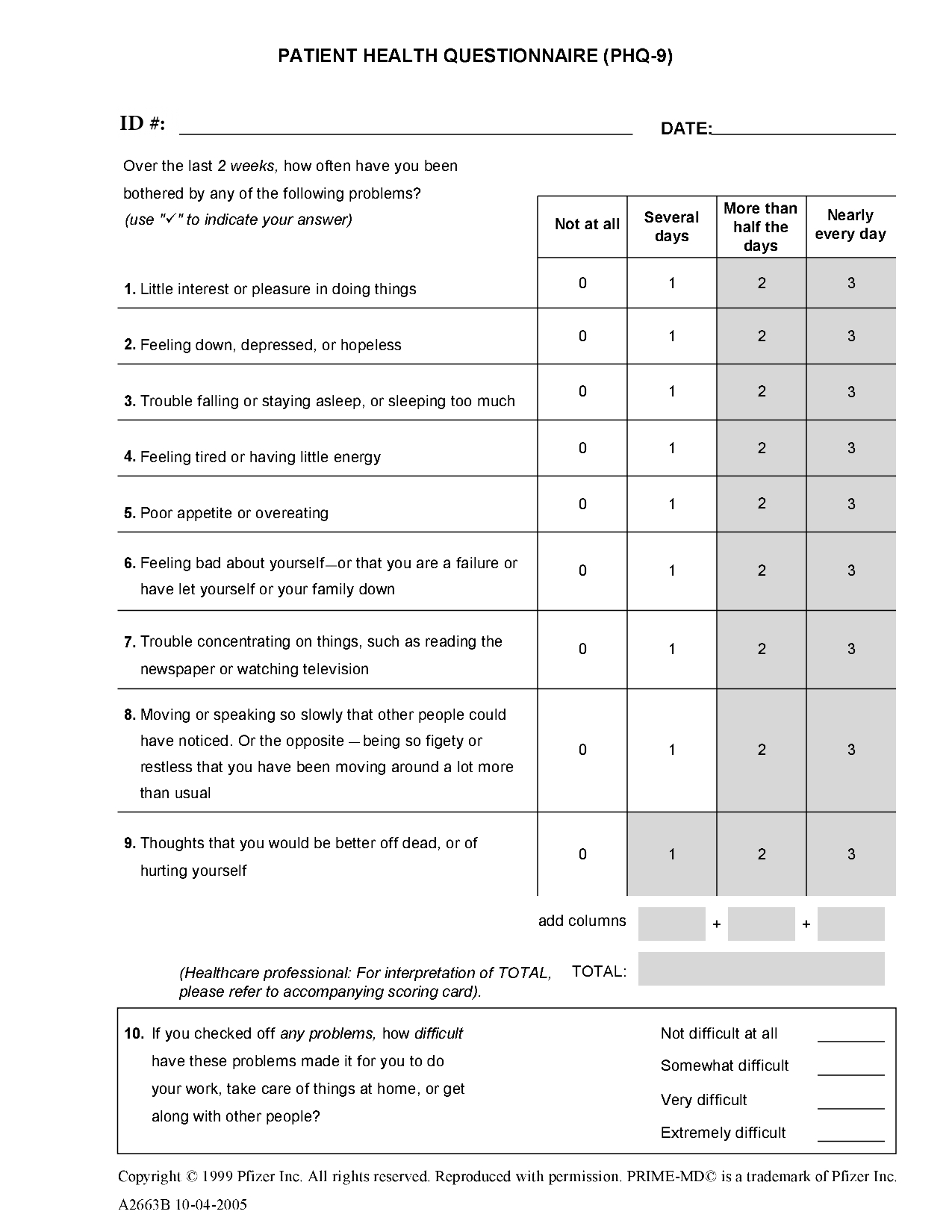


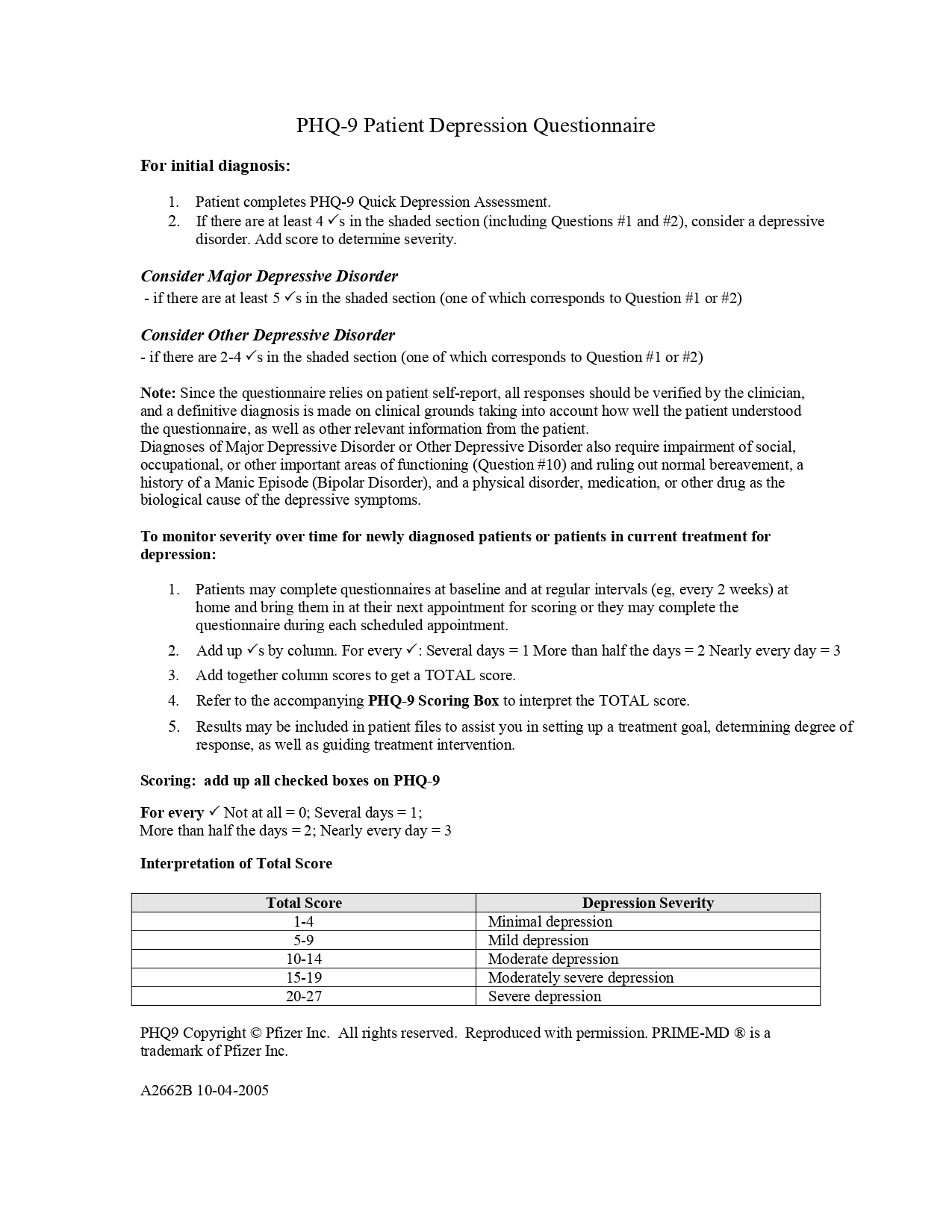

Supplement: Supplementary file 6 [file mmc6.docx]
