# Supplementary material for: Three-year assessment of cognitive and olfactory disturbances among COVID-19 convalescent patients grouped by olfactory hallucination status in Armenia: A qualitative and quantitative study
Source: Clin Med (Lond). 2025 Jul 16;25(5):100489. doi: 10.1016/j.clinme.2025.100489 (PMC12395519; doi:10.1016/j.clinme.2025.100489)
Supplement: Supplementary file 7 — Appendix A. Semi Structured Interview Guide (English & Armenian + Consent Form). [file mmc7.docx]

**Table G.1:** Participant responses to the qualitative questions

| **No.** | **Themes** | **Codes** | **Quotes** |
| --- | --- | --- | --- |
| 1 | Olfactory disturbances | Anosmia | On the third day of Covid, I noticed that I couldn’t feel odors, when I tried to sense odors, I noticed that I didn’t feel them I can’t feel the smell of gas I couldn’t feel bad smells, some species of flowers, the smell of apricots, and the smell of toilets Some odors I couldn’t feel, like coffee, some of them are recovered now, and I can smell them normally, but I still can’t feel some I used a nasal spray that had a strong odor, and now I can’t sense it Sleeping mattress was burning, and the house could’ve burned, that’s when I knew I had lost my sense of smell and taste I stopped feeling the odor of perfumes and was more stressed because of that I lost my smell on the second day of Covid, I had a total loss for eight months I noticed a total loss of smell and taste on the 5th day of the disease On the 5th day, I woke up and could not sense the smell of my perfume, then I suspected that I was infected, and I went to the polyclinic and tested positive I couldn’t feel the pleasant smell of shampoo while showering I couldn’t smell garlic, which I usually get irritated with  I tried some alcohol and oil at home, still couldn’t smell Loss of smell from certain subjects like metallic smells Immediately after quarantine, I lost my smell I could not feel anything in the case of chocolate |
|  |  | Hyposmia | Some I can feel normally, like cinnamon; others I feel mildly, like perfumes Before Covid, I was very sensitive to smells and used to react to even the mildest odors, but now I can't smell strongly When I cut the garlic, I can smell it mildly, and then it disappears on the second or third sniff, and after I leave it aside for 5 minutes and return, I mildly feel it |
|  |  | Parosmia | For me, perfume, food, and the toilet smell the same When I started to feel the smells but couldn’t differentiate, there was no smell or taste that I would feel, but it was similar to its old smell Nothing smells like its real smell, but bananas are a little bit similar to their old smells, home cleaning liquids are not identical to what they used to, perfumes are very different from what they were I couldn’t differentiate the smell of coffee and gasoline, they’re persistent until now Odors like gasoline, perfumes, and ash are different, I sense them, but they’re unpleasant  I couldn’t differentiate between the smells of different greens, vegetables, cinnamon, and cloves during cooking I can smell tea but it’s not the same The only smell I can differentiate is the smell of citrus Perfumes now smell very bad, and each fragrance has a specific unpleasant odor component, the perfumes that I used to use are now unpleasant to me I believe that I am unable to differentiate smells Now the smell of cucumber is significantly expressed, just like perfumes I started differentiating some odors, but they were disturbed, some significantly expressed disturbed smells were meat, greens, vegetables, cucumber, and pepper and coffee tasted disgusting, and the scent of perfumes was unpleasant, coca cola was also undesirable Coffee was not the same for me and was terrible White onions have a disgusting smell for me After Covid, I felt disgusting odors from chocolate, rose, and oranges, they smelled like old dirty socks Roses smell like alcohol, essential oils are disturbed, roses that used to smell like dirty old socks now smell like alcohol Initially, I could still feel solid odors but had an issue with sweet smells |
|  |  | Phantosmia | When when I don’t feel odors, everything smells disgusting somehow Often I was suffocating on the scent of non-existing smoke, even when I couldn’t smell something, I used to suffocate from it |
| 2 | Gustatory disturbances | Ageusia | I loved cucumbers, but now cucumbers are disgusting I also feel a terrible taste from cooked meat and cucumber In the beginning, I couldn’t feel any taste and was eating just for the sake of it I noticed a total loss of smell and taste on the 5th day of the disease I remember how a cucumber smells and tastes, but I don’t feel its taste until today I could feel the primary taste (salty, sour, spicy), but I couldn’t feel the taste of orange I couldn’t taste as well,these changes lasted 1 to 2 months |
|  |  | Hypogeusia | Then I started feeling some flavors, but they were still very mild |
|  |  | Dysgeusia | The most negative aspect is that I had long-term weight loss because I didn’t eat properly for eight months due to disturbed tastes I could feel the sweet, sour, and spicy flavors, but I didn’t feel any specific taste when I ate a salad For sunflower seeds, I can eat them raw, but when they’re cooked or smoked, I can’t, it’s somehow disgusting Coca-cola doesn’t taste like before  Especially pork barbecue that I used to eat, it wasn’t only not tasty, but unpleasant, I didn’t eat pork for five months, and I used to feel it even if I didn’t know if the food had pork I always liked Kyiev-style cutlets with amazing tastes and smells for me, and suddenly it was unpleasant and disgusting, the chicken was also very unpleasant, but I had to eat it |
|  |  | Phantogeusia | I can sense the smell of smoke when I lie down, and it’s very unpleasant and choking, this smell is there when there’s no source of the smoke but it’s not there when I’m in a sitting position I woke up in the middle of the night while choking on a strong smoky smell (like burnt oil or aluminum), I thought something in the kitchen was burning and ran to the kitchen and saw there was nothing, and for 2 days, the smoke smell didn’t go away, I don’t know how to explain the absence of the smoke, but I felt choking |
| 3 | Timeline of chemosensory alteration after COVID-19 diagnosis | Sudden | It happened suddenly when I saw smoke in the house, and I couldn’t smell it All these changes were sudden I think the sweet smells recovered suddenly in my case |
|  |  | Gradual | For a long time, I had a loss of smell for approximately a year I could still smell it in the first week, but then it gradually disappeared I couldn’t feel some odors, but now I can smell them, like cinnamon, coffee, and clover. However, I have to smell them from a close distance and for a long time It started coming back slowly, and I was starting to feel them stronger over time It was gradually recovering as I smelled different stuff  Especially the smell of sweat, I started to slowly feel the smell of sweat that I couldn’t feel, I was happy, but I understood that I was feeling it differently My sense of smell started recovering slowly I didn’t feel anything for two months, I couldn’t feel the pleasant smell of shampoo while showering, then slowly, I could feel it, and now I can fully smell shampoo and gels After one month, recovery started gradually, I used to feel some smells during the daytime and then couldn’t later in the day and his lasted for two months, after which there was good improvement |
|  |  | Non-linear | Sense of smell stayed normal for a couple of days, then it worsened again Smell and taste disturbances were lost 3-4 days after Covid, then, it suddenly recovered two days before my birthday, after which disturbances started First, it was a sudden loss again, and then it gradually got disturbed I was starting to feel chocolate, but it went back to disturbed It started recovering 6-7 months later and fully recovered, then after one year and three months, it started deteriorating again, but not as severe as before |
|  |  | Persistent issues | Smell and taste disturbances persist I have severe allergies for which I have been receiving treatment for a year, it began in march and is still persistent, also, issues regarding the GIT, specifically the pancreas I started recovering, but until now, it’s still disturbed Some recovery has occurred, but some materials stayed with the same disturbed smell My persistent issues are citrus (lime), coffee, the smell and taste of which I don’t feel, and chocolate, chicken, pork, and beef are felt, but they’re not the same as before Until now, the smell of pepper is unbearable, I don’t know how to explain to people how disgusting the scent is to me, it is similar to smells coming from shops that change car oils Some smells remained unpleasant, like flowers, and as for food, I don’t enjoy it anymore After one year, these smells are less expressed, but I can still feel them from time to time, there are also smell disturbances and even loss of some smells |
| 4 | Severity of chemosensory alteration | Mild/Moderate | Since it had a mild course, I thought these complications would not last and disappear with time, but, Covid had a significant impact on my health Nothing related to me has changed because the course of my disease was mild, there were some minor disturbances, but I didn’t approach them seriously, and they didn’t impact me much My disease took a mild course, and I did not believe I was infected with the coronavirus |
|  |  | Intense/Severe | I can say that Covid flips life upside down, at least for me I had a severe course of the disease I had a severe course of the disease with pneumonia, and I was on bed rest for ten days, after which I tried to return to everyday life |
| 5 | Quality of life | Physical impact | I can’t even sit for 3 hours I have GIT disturbances After all this, allergic reactions and stuttering appeared I have pain, nausea, diarrhea or constipation, vomiting, burning I get uncontrollable shivering I don’t have much energy to be physically overwhelmed because I’m always tired, even though my job is not physically demanding, I return home feeling like I’ve done the most challenging work I have problems with my immunity, I get sick very quickly, I feel a general weakness, I have vascular issues in my feet, after Covid, there are pains in my legs and when I wear warm pants, my legs start burning  After Covid, I had high-stress times, and I lost 53 to 49 kilograms during Covid I had anemia due to eating less for a period I had fatigue, alertness-related, and sleeping pattern disturbances, I used to wake up in the night and stay awake till sunrise I also have a decreased appetite and I don’t feel hungry Fatigue has increased, I cannot stand on my feet for long hours doing housework, and now it isn’t easy, also sleepiness is expressed and I even sleep without acknowledging it, I still wake up at 7 am even when I sleep late, I wake up with high energy, but it’s all gone during the evening I get lazy if I am deprived of my work and daily activities or when I’m not busy doing anything My first menstrual cycle after Covid was extremely painful, to an amount that I thought I’d lose consciousness |
|  |  | Emotional impact | I was afraid that having Covid would be a life-challenging experience ending in either survival or death This leads me to think, “what have I done to deserve this?, Why should all this happen to me?”, there are no answers to these questions I am afraid that I will not be able to formulate a sentence, which brings to stress I can’t specifically feel the smell of my children, which I liked to feel a bit longer Those that I want to feel strong, I can’t feel, like the smell of my children, I can feel the smell of my youngest child, but not the other two I was lying down disappointed |
|  |  | Psychological impact | I have always been calm in my career, but now, some things stress me out Now when I work, I don’t see the importance of a diploma or studying It made my nervous system stressed I become so stressed and anxious that I freeze and don’t know what to say, so bad that it becomes a laughing matter I have fatigue, absence of motivation, depression of mood I had severe depression, didn’t want to live, and was thinking I was going mad My motivation, energy, and enthusiasm are decreased, now I don’t even want to do what I’m supposed to, the lack of energy leads to reduced motivation My self-value has dropped, and I even considered myself useless It also affected my psychological state, mainly because of the smell disturbances or loss I have been physically and mentally stressed in the last six months I had a bad course of depression |
|  |  | Cognitive impact | The main problem was the extreme loss of focus and the absence of an inner will I cannot focus. Usually, I solve challenging scenarios easily at work. Now I forget my meeting times and lose focus while working If you ask me now, I can’t remember what I study in a week and a half The neurological state got disturbed after Covid I have weakening of mental processes, difficulty in learning new things I confused words and terminology that I did not forget and commonly use, I get stressed because I can’t recall words, formulate sentences, or express ideas properly I can’t formulate my thoughts and speech correctly, and these disturb life In the morning, cognitive functions are more disturbed, problems that I didn’t have after Covid, focus is more disturbed at night Mental processes deteriorated, before Covid, I used to remember things well, but now I can’t Now I have to read a text twice or three times to comprehend normally, I also think slower about some issue, I forget words that I try to recall, I sometimes stop mid-sentence and forget what I was talking about Even when I used to do fast mathematical calculations, I was getting them wrong I started observing myself, some memory-related issues are present I feel those changes daily, even when I want to remember formulas that I used to remember well, I have to read them Sometimes I can’t even remember a simple noun, like I say, “the eating tool” instead of “fork” In the beginning, for three months, there might have been some memory issues Memory loss, specifically visual memory, was developed in me like losing attention and focus I can’t concentrate on reading a book for long or watching a movie till the end, that’s because I am unable to focus I had some mental tiredness, but I can’t pinpoint if it’s because of this or that I used to forget people’s names and was unable to differentiate my children’s names I also had problems with average calculation, I couldn’t even do simple calculations during the previously conducted test (like subtracting 7 from 100) and I have focus issues, too, I ask the same questions three to four times I have memory deterioration and weakness, I could forget a name of a person momentarily, it’s mainly related to short-term memory |
|  |  | Social impact | I avoid being in public places because smells disturb me and make me nauseated, give me a headache, and even diarrhea Due to the inability to interact with people, my friends are trying to cope with me, when we go out together, they avoid wearing perfumes The smell of other people in public places is horrible I didn’t want to go out and participate in happy events |
|  |  | Lifestyle impact | Nowadays, I am unable to complete the simplest of tasks even when putting in too much effort Previously I thought that they’d improve, but now, after a long time, I must accept those changes and adapt to them in everyday life In the beginning, it was so bad that I wanted to throw up, but now I am used to that bad taste of cucumber, and I can eat it I now drink only one type of coffee because the majority of coffee smell like smoke after being prepared Simple stuff like potato and pasta, I couldn’t eat pork or beef for a long time, the only thing I could eat was chicken I couldn’t even eat vegetables like cucumbers since they were very unpleasant I don’t know whether I am now used to the changed forms of these smells or if my senses are improving I can eat meat, but it’s not the same, I used to smell barbecue and taste it, but now I don’t feel the satisfaction or pleasure when smelling or eating barbecued meat well Currently, I prefer to take vegetarian diet to eliminate the risk of food-poisoning, I am avoiding meats and am using yogurts and vegetables more |
|  |  |  |  |
| 6 | Coping mechanism for chemosensory alteration | Prescriptions and Medical treatments | I’ve only been going to treatments for the last three years, no single specialist left that I haven’t visited to recover the quality of my life, but nothing is changing I went and received medication from the neurologist, and after this treatment, I started feeling the smells The specialist at the polyclinic told me that the antibiotic was needed and that I should take pills Whatever they prescribed to me such as Vitamin D, and I didn’t take any antibiotics |
|  |  | Self-medication and lifestyle modifications | I saw a Russian TV report encouraging olfactory training, and I tried to smell fruits to remember them They didn’t prescribe it, I took mild antibiotics fearing weakness I took paracetamol and then ibuprofen for two days I was performing olfactory training as much as possible, and in response to that, I started feeling sweet odors, which were mainly affected |
| 7 | Perceptions of chemosensory alterations pre and post Covid | Olfactory alterations | Smell disturbance transforms a person into a non-efficient person, and these last years were equivalent to not living Even the odors I can smell are not the same as before getting sick I didn’t notice any positive change During Covid, my sense of smell was terrible No problems earlier, never, I’ve always had a strong sense of smell before Covid I could eat homegrown peppers that were not sprayed with harmful chemicals, neither the taste nor the smell was terrible, I associate that with the small number of toxic chemicals, but when I go to the shop, I can’t even pass next to peppers because I feel disgusted, and I think it’s because of the toxic chemicals, I can eat grilled and cooked peppers, maybe because those chemicals evaporate or something changes after processing  If before Covid, I used to sense them stronger, now they’re mild, and I have to think to differentiate |
|  |  | Gustatory alterations | I used to drink an entire bottle of vodka and not feel it I never felt such taste disturbances when stressed or sick before Covid  During Covid, I had a loss of smell and taste, and that’s how I knew I had Covid. It stayed for 1 to 2 months, then those senses recovered, but differently I used to feel specific tastes when eating in general or eating my favorite foods, and then they didn’t taste the same |
|  |  | Other subjective alterations | Some pains were present during Covid but became more severe after the vaccination, I think the allergy and GIT-related issues are because of the vaccine, if I didn’t receive it, they would disappear shortly after Covid I also started to get ill in shorter intervals, before, it was once or twice per year, now more than four times in spring alone |
